# UPLC-LTQ-Orbitrap-Based Cell Metabolomics and Network Pharmacology Analysis to Reveal the Potential Antiarthritic Effects of Pristimerin: In Vitro, In Silico and In Vivo Study

**DOI:** 10.3390/metabo12090839

**Published:** 2022-09-05

**Authors:** Mengying Lv, Qiaoling Liang, Zhaoyong Luo, Bo Han, Tengyang Ni, Yang Wang, Li Tao, Weiting Lyu, Jie Xiang, Yanqing Liu

**Affiliations:** 1 Department of Pharmacy, Institute of Translational Medicine, Medical College, Yangzhou University, Yangzhou 225001, China; 2 The Key Laboratory of Syndrome Differentiation and Treatment of Gastric Cancer of the State Administration of Traditional Chinese Medicine, Yangzhou 225001, China; 3 Key Laboratory of Xinjiang Phytomedicine Resource and Utilization, School of Pharmacy, Ministry of Education, Shihezi University, Shihezi 832002, China; 4 Department of Medicinal Chemistry, Rutgers University, Piscataway, NJ 08854, USA

**Keywords:** pristimerin, rheumatoid arthritis, cell metabolomics, network pharmacology analysis, MH7A, adjuvant-induced arthritis

## Abstract

Rheumatoid arthritis (RA) is characterized by systemic inflammation and synovial hyperplasia. Pristimerin, a natural triterpenoid isolated from plants belonging to the Celastraceae and Hippocrateaceae families, has been reported to exhibit anti-inflammation and anti-proliferation activities. Our study aims to reveal the antiarthritic effects of pristimerin and explore its potential mechanism using in vitro, in silico, and in vivo methods. In the present study, pristimerin treatment led to a dose-dependent decrease in cell viability and migration in TNF-α stimulated human rheumatoid arthritis fibroblast-like synoviocytes MH7A. Moreover, UPLC-LTQ-Orbitrap-based cell metabolomics analysis demonstrated that phospholipid biosynthesis, fatty acid biosynthesis, glutathione metabolism and amino acid metabolic pathways were involved in TNF-α induced MH7A cells after pristimerin treatment. In addition, the adjuvant–induced arthritis (AIA) rat model was employed, and the results exhibited that pristimerin could effectively relieve arthritis symptoms and histopathological damage as well as reduce serum levels of TNF-α, NO and synovial expressions of p-Akt and p-Erk in AIA rats. Furthermore, network pharmacology analysis was performed to visualize crucial protein targets of pristimerin for RA treatment, which showed that the effects were mediated through the MAPK/Erk1/2, PI3K/Akt pathways and directing binding with TNF-α. Taken together, our study not only offered new insights into the biochemical mechanism of natural compounds for RA treatment, but also provided a strategy that integrated in vitro, in silico and in vivo studies to facilitate screening of new anti-RA drugs.

## 1. Introduction

Rheumatoid arthritis (RA) is a chronic autoimmune disease characterized by systemic inflammation and synovial hyperplasia that mainly results from abnormal proliferation of fibroblast-like synoviocytes (FLS) [1]. The worldwide prevalence of RA from 1980 to 2019 affects almost 1% of the population [2]. It has been clarified that due to the stressful microenvironment of inflamed tissues, metabolic changes in synovial fibroblasts occur and resetting metabolism may offer novel opportunities for disease modulation in RA [3]. Therefore, efficacy evaluation of clinical drugs and new drug candidates focusing on cell metabolomics-based study is needed to uncover metabolic responses of RA-FLS after treatment.

**Figure 1:**
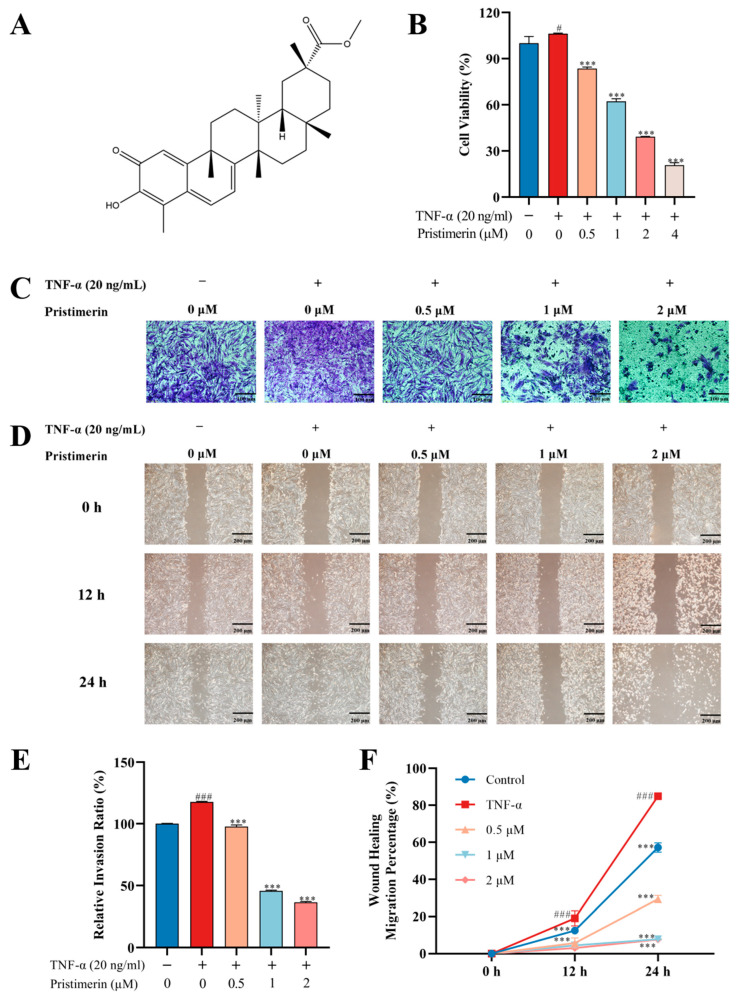
Effects of pristimerin on cell viability and migration. (**A**) Chemical structure of pristimerin; (**B**) MH7A cells were treated with pristimerin (0.5, 1, 2 μM) on MH7A cells for 24 h. Cell viability was determined by MTT assay; (**C**,**E**) MH7A cells were incubated with pristimerin (0.5, 1 and 2 μM) for 24 h. The migrated cells were stained with crystal violet and the relative cell migration was calculated. (**D**,**F**) For the wound healing assay, artificial wounds were created in MH7A cells, which were then treated with pristimerin (0.5, 1, and 2 μM). The migrated distance was determined at 0, 12, 24 h and represented as migration rate. *** *p* < 0.001 compared with TNF-α group. # *p* < 0.05 compared with CON group; ### *p* < 0.001 compared with CON group.

Natural compounds offer a valuable repository of potential therapeutic drugs for incurable and chronic diseases. Pristimerin (Figure 1A) is a bioactive triterpene mainly discovered from species belonging to the Celastraceae and Hippocrateaceae families [4,5]. Numerous studies revealed that pristimerin possesses anti-cancer, anti-inflammatory, anti-oxidant, anti-microbial and insecticidal activities. It has been reported that pristimerin exhibited antiarthritic effect through inhibiting angiogenesis, inflammation, cartilage damage and bone erosion [6,7,8]. Recently, several research studies have focused on the potential treatment of natural triterpenoid compounds for RA therapy. Celastrol, a quinone-methylated triterpenoid extracted from *Tripterygium wilfordii*, can relieve RA symptoms and inhibit inflammation by inhibiting the ROS-NF-κB-NLRP3 axis [9]. The inhibitory effects of Antcin K, a triterpenoid isolated from *A. cinnamomea*, upon TNF-α, IL-1β and IL-8 expression in human rheumatoid synovial fibroblasts was achieved through the downregulation of the FAK, PI3K, AKT and NF-κB signaling cascades [10]. However, the literature capturing subtle changes in cellular metabolism after exposure to natural-derived candidates, including triterpenoids, in anti-RA new drug discovery pipeline, is limited.

Metabolomics, which focuses on simultaneous determination of all low-molecular-weight compounds (e.g., metabolites, the end products of physiological processes influenced by endogenous or exogenous stimuli) in a biological sample, is thought to better represent the disease phenotype [11,12]. Currently, cell metabolomics has been increasingly used to address essential questions in the biomedical field [13]. During rheumatoid synovial inflammation, RA-FLS are the key drivers of synovial hyperplasia and joint destruction as the activated FLS would abnormally proliferate, migrate and finally attach to the adjacent cartilage, forming cartilage–pannus junction and leading to joint destruction, therefore increasing attention has been paid to RA-FLS as an attractive therapeutic target [14,15]. The activated FLS suffers from changes in proto-oncogene expression and signaling pathways, which could trigger downstream metabolic alterations in synovial cells and tissues [16]. Thus, apart from anti-inflammation evaluation, the cell metabolomics-based study is essential to obtain better understanding for analyzing the underling mechanism of pristimerin on RA treatment.

In this study, a strategy that integrated in vitro, in silico and in vivo studies was utilized to reveal the potential antiarthritic effects of pristimerin. Firstly, TNF-α stimulated human rheumatoid arthritis fibroblast-like synoviocyte MH7A cells were applied to test the effects of pristimerin on proliferation and migration using MTT and transwell assays. Moreover, crucial protein targets of pristimerin were determined by Western blot analysis. Subsequently, ultra-high performance liquid chromatography (UPLC)-LTQ-Orbitrap-based untargeted metabolomics approach was employed to reveal pristimerin-induced metabolic changes in MH7A cells. Finally, combined with network pharmacology analysis, the adjuvant–induced arthritis (AIA) rat model was used to study in vivo anti-arthritic activity of pristimerin and evaluate the potential mechanism. Our study is expected to provide new insights into the antiarthritis mechanism of pristimerin and lay foundation for discovering new anti-RA drug targeting RA-FLS and metabolic pathways.

## 2. Materials and Methods

### 2.1. Effects of Pristimerin on TNF-α-Stimulated MH7A Cells

#### 2.1.1. Cell Culture

Human rheumatoid arthritis fibroblast-like synovial cell line MH7A was supplied by Beina Biological Company (Beijing, China). Cells were cultured in DMEM supplemented with 15% fetal bovine serum (Gibco) and 1% penicillin-streptomycin (Gibco) at a 37 °C, 5% CO_2_ incubator.

#### 2.1.2. Cell Viability Assay

3-(4,5-dimethylthiazoyl-2-yl)-2,5-diphenyltetrazolium bromide (MTT) assay was used to measure viability of MH7A cells, which were seeded in 96-well plates (5 × 10^3^/well) and cultured for 24 h. Cells were stimulated by TNF-α (20 ng/mL) and treated with different concentrations of pristimerin (0.5, 1, and 2 μM) for 24 h. Then, 20 μL MTT was added to each well and incubated for another 4 h. Subsequently, 150 μL DMSO was used to replace the medium and the plate was gently shaken for 10 min. The absorbance value of each well was determined at 490 nm using an EnSpire multimode plate reader (PerkinElmer).

#### 2.1.3. Transwell and Wound Healing Migration Assay

Transwell chambers (Corning) were used to assess the effects of pristimerin on cell migration. MH7A cells were seeded in upper chambers with TNF-α (20 ng/mL) and pristimerin (0.5, 1, and 2 μM). Meanwhile, 600 μL of medium with 10% FBS was added to lower chambers. Twenty-four hours later, cells were fixed with 4% paraformaldehyde for 30 min and stained with crystal violet for 20 min.

In wound healing assay, MH7A cells were seeded in 6-well plates for 24 h. When MH7A cells reaching 90% confluency, artificial wounds were created. After stimulation by TNF-α (20 ng/mL) and treatment with pristimerin (0.5, 1, and 2 μM), the distance of cell migration at 0, 12, and 24 h was observed using an inverted microscope.

#### 2.1.4. Cell Metabolomics

- Sample preparation:

MH7A cells were cultured as described above and fixed with 1 mL ice-cold methanol after being washed three times with PBS [17]. The cells were scraped from plates with a cell scraper and the cell-methanol mixture was subjected to 3 freeze and thaw cycles: firstly, freeze in liquid nitrogen for 5 min and then thaw at room temperature for 5 min [18]. Subsequent to centrifugation at 13,000× *g* for 20 min at 4 °C, supernatants were collected and evaporated under nitrogen at room temperature. The cell extract was resuspended in 60 μL methanol, and 5 μL was used for UPLC-LTQ-Orbitrap-MS-based metabolome assay. Additionally, quality control (QC) samples were prepared by mixing equal volume of each sample to guarantee the reproducibility and reliability of the analytical method.

- UPLC-LTQ-Orbitrap-MS analysis:

Profiles of cell metabolomes were obtained by an UPLC system (Dionex, Thermo Fisher Scientific; Sunnyvale, CA, USA) coupled to an LTQ-Orbitrap Elite mass spectrometer (Thermo Fisher Scientific; Bremen, Germany) using an electrospray ionization (ESI) source in both positive and negative ion modes. The chromatographic separation was achieved on an ACQUITY UPLC BEH C18 column (150 mm × 2.1 mm, 1.7 μm) (Waters, Milford, MA, USA) at 35 °C. Mobile phase A was water with 0.1% formic acid and mobile phase B was acetonitrile with 0.1% formic acid. The flow rate was set at 0.4 mL/min and the mobile phase B increased linearly from 10% to 40% in 2 min at first, then directly raised to 80% within 5 min. After a linear increase to 90% in 4 min and maintaining for 4 min, the mobile phase B was restored to 10% for equilibration. Parameters for mass spectrometer were set as follows: spray voltage: 3.8 kV for positive mode and 3.2 kV for negative mode; capillary and heater temperature: 350 °C; sheath gas flow rate: 45 psi; auxiliary gas flow rate: 15 psi; S-Lens RF level (%), 60 and scan range (*m*/*z*):50–1000.

- Data processing and potential biomarker screening:

The raw UPLC-LTQ-Orbitrap-MS data were processed by Compound Discoverer 2.1 (Thermo Fisher Scientific, Waltham, MA, USA) and then the data were subjected to background deduction, “30% rule in QC samples” and total area normalization, as previously described [19]. Then the preprocessed data were further analyzed by SIMCA-P (Umetrics, Umeå, Sweden, Version 13.0) to perform principal components analysis (PCA) and orthogonal partial least squares discriminant analysis (OPLS-DA). Differential variables that met a variable importance in projection (VIP) value > 1.0 and *p* value (Mann–Whitney test) < 0.05 were retained for structural annotation.

Potential biomarkers were annotated by comparing chromatographic and mass information with that of standard compounds or data searched in databases such as Human Metabolome Database, PubChem Compound, ChemSpider and MassBank. The changes of potential biomarkers in all test samples were presented in a heatmap. MetaboAnalyst 5.0 (https://www.metaboanalyst.ca/, accessed on 27 May 2022) was used for metabolic pathway analysis and the metabolic network of potential biomarkers were constructed according to their relationships in KEGG and related literatures [20].

#### 2.1.5. Network Pharmacology Analysis

RA-associated targets were collected from GeneCards (https://www.genecards.org, accessed on 27 May 2022) and DisGeNET (https://www.disgenet.org, accessed on 27 May 2022) databases using the keyword “rheumatoid arthritis” and pristimerin-related targets were obtained by searching against the literature and databases such as Swiss Target Prediction (http://www.swisstargetprediction.ch/index.php, accessed on 27 May 2022) and GeneCards. The overlapped genes were regarded as the candidate targets of pristimerin for RA treatment and were used as an initial input in STRING (https://www.string-db.org, accessed on 27 May 2022) to construct a protein–protein interaction (PPI) network. MATLAB 2020b was utilized to analyze the network and topological features including degree, eigenvector centrality, betweenness and closeness were calculated to identify key targets, which were used to perform Gene KEGG pathway enrichment analysis.

#### 2.1.6. Western Blotting Assay

Following TNF-α stimulation and pristimerin treatments, MH7A cells were washed with ice-cold PBS and lysed with RIPA buffer (Beyotime Institute of Biotechnology, Nanjing, China). Cell debris were removed by centrifugation at 12,000× *g* at 4 °C and the bicinchoninic acid (BCA) protein assay kit (Beyotime Institute of Biotechnology, Nanjing, China) was used for protein quantification. Equal amount of protein was separated by sodium dodecyl sulfate-polyacrylamide gel electrophoresis (SDS-PAGE) and transferred onto PVDF membranes. After blocking with 5% skim milk in TBST buffer for 2 h, the membranes were incubated with primary antibodies including p-Akt, Akt, p-Erk1/2 and Erk1/2. After washing with TBST buffer, the membranes were incubated with HRP-conjugated anti-rabbit second antibody. Subsequent to washing and enhanced chemiluminescence (ECL, Bio-Rad, cat. no.170-5061), protein bands were visualized by a ChemiDoc XRS system (Bio-Rad).

#### 2.1.7. Molecular Docking Study

The chemical structure of pristimerin was obtained from the Pubchem compound database (http://pubchem.ncbi.nlm.nih.gov, accessed on 27 May 2022) with ID as 1258-84-0. The crystal structure of TNF-α protein with PDB IDs as 2AZ5 [21] was obtained from the Research Collaboratory for Structural Bioinformatics (RCSB) Protein Data Bank. The crystal water molecules, ligand atoms, and ions that were bound to the proteins were removed. Hydrogen atoms were subsequently added using AutoDock Tool (ADT) program, version 1.5.6. Molecular dockings between pristimerin and predicted target proteins were performed using AutoDock Vina, based on the Lamarckian genetic algorithm, which combines energy evaluation through grids of affinity potential to find a suitable binding position for a ligand on a given protein. The grid box for docking was positioned properly at the active binding site in the center. The genetic algorithm and its run were set to 1000, as the docking algorithms were set on default. Finally, results were retrieved as binding energy and docking with binding energies lower than −5 kcal/mol was selected as significant binding event and was visualized using PyMol version 1.5.0.3. software for obtaining hydrogen bond, hydrophobic, and electrostatic interactions [22].

### 2.2. Effects of Pristimerin on Adjuvant-Induced Arthritis (AIA) Rats

#### 2.2.1. AIA Rat Model and Experimental Protocols

Male Wistar rats were purchased from the Comparative Medicine Centre of Yangzhou University (Yangzhou, China). The experiment was approved by Institutional Animal Care and Use Committee of Yangzhou University and all the procedures were in accordance with China laboratory regulation Act (2017) under a Project License (SYXK(SU)2017-0044). The rats were in a temperature (22 ± 2 °C)-controlled environment and had free access to food and water.

Twenty-four male Wistar rats (eight weeks old) were randomly divided into four groups: control group (CON), AIA group (AIA), AIA + Pristimerin (PRI) and the AIA + methotrexate (MTX) group, with six animals per group. After adaptation for one week, the AIA model was utilized according to our previous research [23]. The next day after complete Freund’s adjuvant (CFA) induction, rats in the PRI group were intragastrically administered with pristimerin (0.8 mg/kg/d, dissolved in 0.3% CMC-Na) for consecutive 28 days and rats in the MTX group were treated with MTX (0.6 mg/kg/w). For the rats in the CON and AIA groups, they were given 0.3% CMC-Na. During the drug treatment period, the paw volume of the rats was measured and the arthritis scores were evaluated after CFA induction based on a five-grade scoring system [24]. On day 28 after CFA immunization, the rats were anesthetized with chloral hydrate and blood samples were collected using abdominal aortic method. After centrifugation at 1200× *g* for 10 min, serum was collected and were used for quantification of TNF-α and NO by the commercial ELISA kit (Tongwei Co. Ltd., Shanghai, China) and NO assay kit (Nanjing Jiancheng Bioengineering Institute, Nanjing, China) based on the manufacturer’s instructions.

#### 2.2.2. Histopathological Examination and Immunohistochemical Examination of Ankle Joints

The right ankle joints were removed, skinned and fixed in 4% formaldehyde solution for histopathological and immunohistochemical analysis. After decalcification in 30% formic acid for 14 days at 4 °C, they were embedded in paraffin wax and cut into sections about 4 μm in thickness. The sections were stained with hematoxylin-eosin (H & E) and observed by light microscopy. In the meanwhile, the antibodies against p-Akt and p-Erk were analyzed in ankle joint tissues and the positive expression colors were brown or yellow [25].

### 2.3. Statistical Analysis

Data were expressed as mean ± standard deviation (SD) and analyzed via one-way analysis of variance (ANOVA), and *p* < 0.05 was considered as a significant difference.

## 3. Results

### 3.1. Effects of Pristimerin on TNF-α-Stimulated MH7A Cells

#### 3.1.1. Inhibition of Cell Viability and Migration by Pristimerin in TNF-α-Stimulated MH7A Cells

To evaluate the effects of pristimerin on cell viability, TNF-α stimulated MH7A cells were treated with different concentrations of pristimerin for 24 h, and cell viability was determined by MTT. As shown in (Figure 1B), pristimerin treatment led to a dose-dependent decrease in cell viability in TNF-α stimulated MH7A cells. Inhibitory concentration (IC) 50 values after exposure to pristimerin for 24 h were 1.408 μM. Transwell (Figure 1C,E) and wound healing assays (Figure 1D,F) revealed that the number of migrated cells was significantly decreased after pristimerin treatment. Moreover, this migration inhibition ability of pristimerin showed a dose-dependent manner.

#### 3.1.2. Metabolic Regulation by Pristimerin in TNF-α-Stimulated MH7A Cells

**Figure 2:**
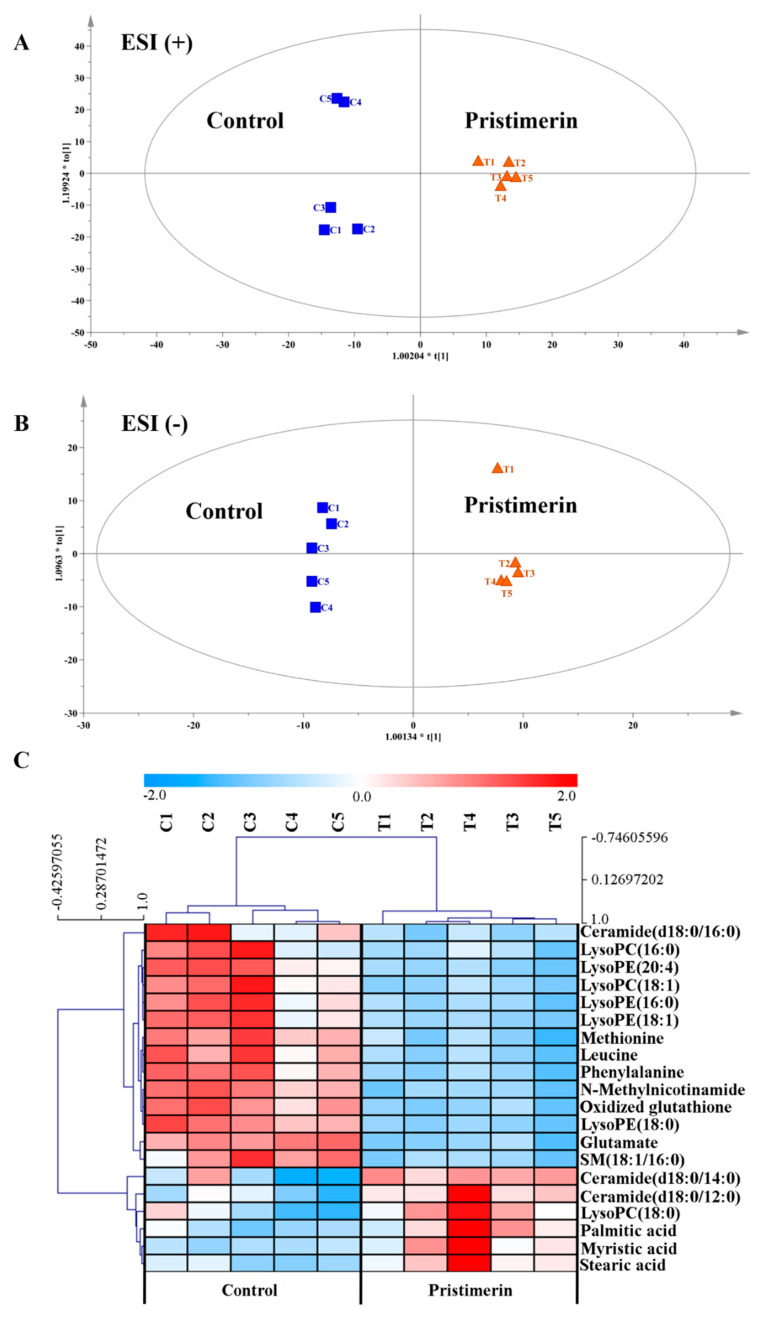
Metabolic effects of pristimerin on TNF-α-stimulated MH7A cells. OPLS-DA models of the untreated group vs. pristimerin-treated group in the positive (**A**) and negative (**B**) mode; (**C**) Hierarchical clustering heat-map analysis of metabolic changes in TNF-α-stimulated MH7A cells after pristimerin exposure, *n* = 5 per group.

**Figure 3:**
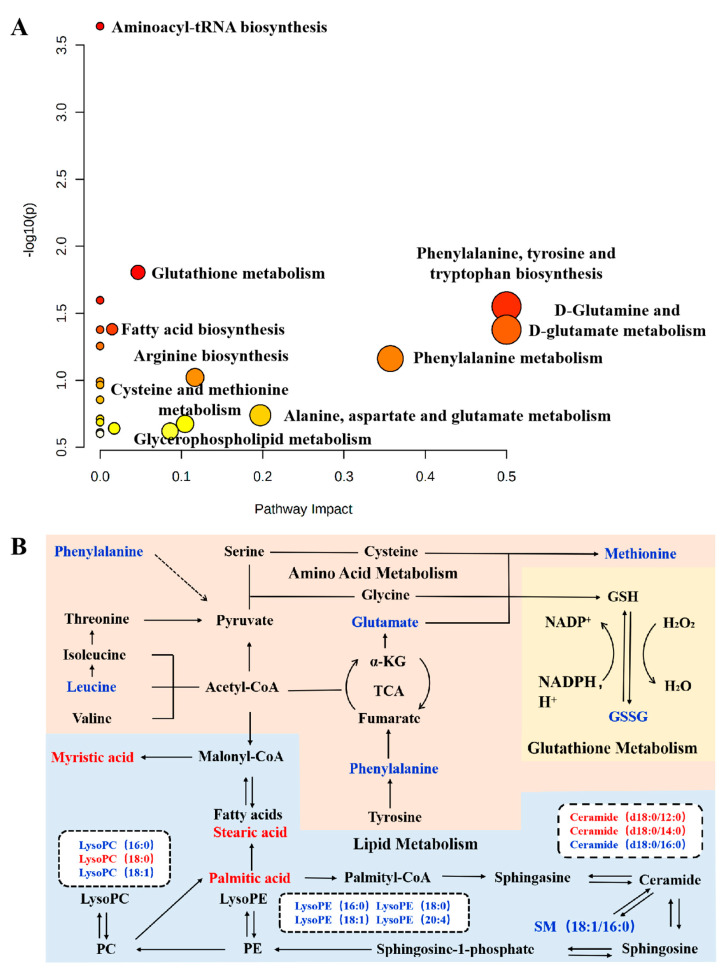
(**A**) Metabolic pathway analysis using Mataboanalyst 5.0; (**B**) Schematic diagram of the altered metabolites and potential disturbed metabolic pathways. Up-regulated metabolites detected are shown in red; down-regulated metabolites detected are shown in the blue. Abbreviations: α-KG, alpha-ketoglutaric acid; PC, phosphatidylcholine; PE, phosphatidylethanolamine; TCA, tricarboxylic acid; SM, sphingomyelin.

To capture the subtle metabolic alterations in TNF-α-stimulated MH7A cells after pristimerin treatment, an untargeted metabolomics analysis using UPLC-LTQ-Orbitrap-MS was performed and we found that compared with the untreated group, the endo-metabolome of MH7A cells was significantly changed after exposure to pristimerin. The representative chromatograms were shown in Figure S1. PCA (Figure S2) and OPLS-DA (Figure 2A,B) models were established using SIMCA-P and a clear separation between the untreated group and pristimerin-treated group was observed in positive and negative ionization modes. VIP values of each variable were calculated from the OPLS-DA model and were used to identify variables that contributed greatly to group separation. Moreover, nonparametric (Wilcoxon, Mann–Whitney) test was performed to determine the significance of each variable and variables VIP > 1.0 and *p* < 0.05 were screened out. After structural annotation, 20 differential metabolites were discovered in pristimerin-treated cells and the heatmap (Figure 2C) was used to visualize their relative concentration variations in each sample. The detailed information of these potential biomarkers was presented in Table S1. MetaboAnalyst 5.0 software was used to identify which pathways were disturbed by pristimerin [20]. Multiple metabolic pathways especially phospholipid biosynthesis, fatty acid biosynthesis, glutathione metabolism and amino acid metabolism were found to be significantly perturbed, indicating that these metabolic pathways were involved in pristimerin-treated MH7A cells. Furthermore, we observed a significant decrease in oxidized glutathione, many amino acids and lysophospholipids such as lysophosphatidylethanolamines (LysoPEs) and lysophosphatidylcholines (LysoPCs) and a marked increase in certain fatty acids and ceramides. The schematic diagram of the altered metabolites and a more specific elucidation of these metabolites was presented in Figure 3.

#### 3.1.3. Potential Targets of Pristimerin for RA Treatment

996 RA-associated targets were collected after combining information from two databases, in which 847 targets (relevance score ≥ 5) were obtained from the GeneCards and 777 targets (GDA score ≥ 0.1) were acquired from DisGeNET. One hundred and thirty pristimerin-related targets were gathered from the literature and databases such as Swiss Target Prediction and GeneCards. After a comparison between RA-associated genes and pristimerin-related targets, 50 overlapped targets were considered as candidate targets of pristimerin for RA treatment and were used to construct a protein–protein interaction (PPI) network using STRING. The PPI network was assessed by topological features including degree, eigenvector centrality, betweenness and closeness using Matlab and 23 targets were regarded as crucial for the antiarthritic mechanism of pristimerin (Figure 3A). Kyoto Encyclopedia of Genes and Genomes (KEGG) pathway enrichment analysis (Figure 3B) revealed that these 23 crucial targets were mainly involved in TNF signaling pathway, PI3K-Akt signaling pathway.

#### 3.1.4. Inhibition of p-Akt and p-Erk Expressions by Pristimerin in TNF-α-Stimulated MH7A Cells

**Figure 4:**
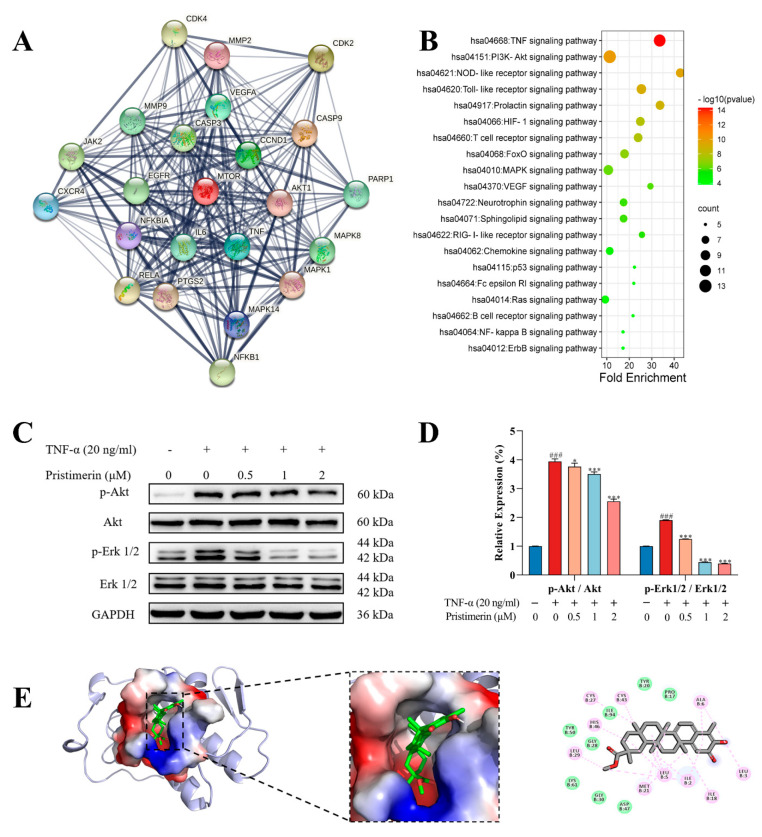
(**A**)The PPI network based on 23 potential key targets of pristimerin for RA; (**B**) The bubble chart of KEGG enrichment based on 23 potential key targets. (**C**,**D**) Effects of pristimerin on MAPK/Erk1/2 and PI3K/Akt pathways. Glyceraldehyde-3-phosphate dehydrogenase (GAPDH) was used as an internal control. Results were presented as the mean ± standard derivations in Western blotting assay. (**E**) Binding of TNF-α with pristimerin and the inset showing the principal interactive residues of pristimerin at the TNF-α binding pocket. * *p* < 0.05 compared with TNF-α group; *** *p* < 0.001 compared with TNF-α group, ### *p* < 0.001 compared with CON group.

To determine the impact of pristimerin on MAPK/Erk1/2 and PI3K/Akt pathways in MH7A cells, the expression of p-Erk1/2, Erk1/2, p-Akt and Akt was measured by Western blotting. Our results (Figure 4C,D) revealed that pristimerin treatment inhibited Erk1/2 and Akt activation through dose-dependently decrease the expression of p-Erk1/2 and p-Akt without changes in total Erk1/2 and Akt levels. Overall, these data demonstrated that pristimein inhibited MH7A cell proliferation and migration through suppressing Erk1/2 and Akt activation.

#### 3.1.5. Interaction of Pristimerin with Potential Targets Regarding Metabolic Pathways

The molecular docking study was conducted using AutoDock Vina 4.2.6. The binding affinity and the presence of hydrogen bonds and hydrophobic bonds are predicted (Table S2). Molecular docking analysis shows that pristimerin has an interactive affinity simulation −9.5 kcal/mol) with the active site of TNF-α. The interactions between pristimerin and TNF-α are identified. As shown in Figure 4E, they are eight van der Waals with Leu 120, Gly121, Tyr59, Gly121, His15, Tyr119, Ser60, and Tyr151, two π-alkyl stacking interaction with Tyr119 and Ile 155 and a π-π stacking with Tyr59.

### 3.2. Therapeutic Effects of Pristimerin on AIA Rats

#### 3.2.1. Effects of Pristimerin on Degree of Paw Swelling, Arthritis Scores and Serum Levels of TNF-α and NO

**Figure 5:**
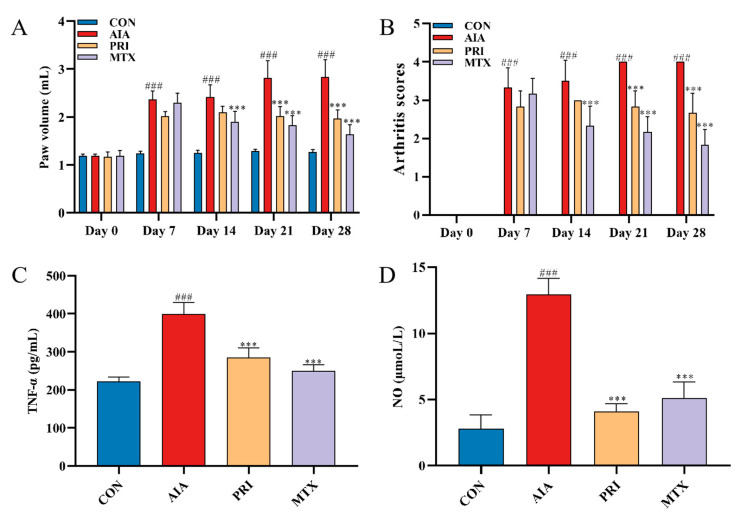
Effects of PRI on paw swelling (**A**), arthritis scores (**B**), serum levels of TNF-α (**C**) and NO (**D**) of AIA rats. All the data were expressed as mean ±SD, *** *p* < 0.001 compared with AIA group, ### *p* < 0.001 compared with CON group.

As shown in Figure 5A,B, there was an obvious increase in the right hind primary paw volume in the AIA group. PRI and MTX treatment significantly decreased the primary paw volume and arthritis scores after CFA induction. Compared with the CON group, the serum levels of TNF-α and NO were upregulated in the AIA group. However, PRI and MTX administration could significantly inhibit the CFA-induced TNF-α generation and NO production (Figure 5C,D).

#### 3.2.2. Effects of Pristimerin on Histopathological Changes of Ankle Joints and Immunochemical Expression of p-Akt and p-Erk

**Figure 6:**
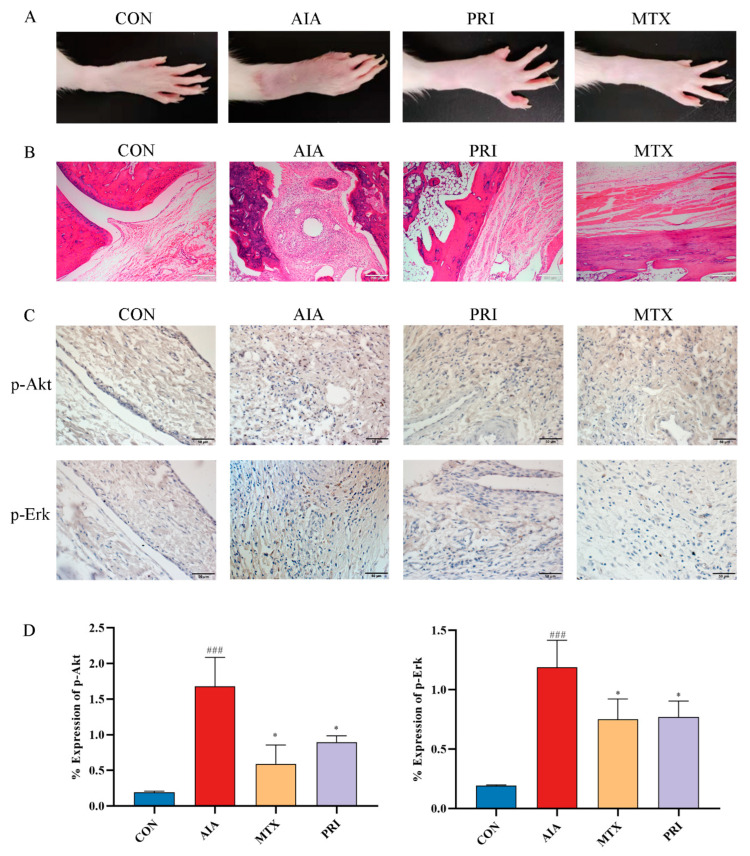
Typical photos of PRI effects on right hind paws of rats (**A**), histopathological changes in ankle joints (**B**, ×100, HE staining) and the immunohistochemical expression of anti-p-Akt and anti-p-Erk antibodies in ankle joints (**C**, ×400, **D**), * *p* < 0.05 compared with AIA group, ### *p* < 0.001 compared with CON group.

As can be seen from Figure 6A, the rat’s right hind paw was significantly swollen in the AIA group, which were alleviated by treatment with PRI and MTX. Furthermore, the anti-RA effects of PRI in AIA rats was examined through H&E staining (Figure 6B). Obvious infiltration of inflammatory cells and bone erosion was observed in AIA rats and these RA symptoms were significantly alleviated after treatment with PRI and MTX. Moreover, the expressions of p-Akt and p-Erk in ankle joints of rats were analyzed by immunohistochemistry method (Figure 6C,D). Reduction in p-Akt and p-Erk levels in AIA rats was observed after treatments with PRI and MTX.

## 4. Discussion

RA is characterized by chronic inflammation of synovial tissue and progressive destruction of cartilage and bone, which is defined as the most common autoimmune, destructive, inflammatory arthritis in adults [26]. Without immediate and appropriate treatment, the pathological process of patients may lead to joint deformity and disability. Currently, commonly used agents for the RA treatment include non-steroidal anti-inflammatory drugs (NSAIDs), disease modifying anti-rheumatic drugs (DMARDs), glucocorticoids and biological agents [27,28]. However, some unanticipated adverse effects occasionally occurred during the long therapeutic procedure: we were expecting urgent adjuvant therapy to be available in clinical practice, especially the potential targets related to metabolic pathways [29,30].

Pristimerin, a quinone-methyl triterpenoid isolated from the Celastraceae and Hippocrateaceae families, has shown anti-tumor, anti-inflammatory and antimicrobial activities by regulating multiple signaling pathways [31,32]. Pristimerin is a methyl ester of celastrol. Recently, more researchers have focused on the potential treatment of celastrol for lipid-induced metabolic dysfunctions and obesity [33,34]. It has been well clarified that serum lipid profiles was improved after celastrol co-supplementation in the HFD-fed mice [35] and celastrol was evaluated to be able to modulate lipid metabolism [36] and inhibit adipocyte differentiation [37]. Up to now, the anti-RA potential of pristimerin is mostly confined to the anti-inflammation activity upon tissue damage alleviation and its functional potential on metabolic modulation are rarely mentioned.

Synovial hyperplasia plays an important role in the pathological progression of RA and RA-FLS, as the dominant cell type comprising the hyperplastic synovium, are the main culprits in joint deformity and destruction. They are characterized by a tumor-like aggressive phenotype. The abnormally proliferated RA-FLS would migrate and invade into the cartilage. Accordingly, targeting RA-FLS and inhibiting its proliferation and migration has emerged as an efficient therapeutic approach for RA [38]. TNF-α-induced human RA synovial cell line of MH7A have been widely used to screen potential anti-RA compounds from herbal medicines [39]. In this study, the potential antiarthritic effects of pristimerin on human rheumatoid fibroblast-like synoviocytes MH7A were investigated through phenotypic assays, Western blot analysis, and cell metabolomics. For preclinical screening of anti-RA drugs, various methods have been employed to induce arthritis in experimental animals, among which AIA and collagen-induced arthritis animal models are extensively used [40]. PI3K/Akt signaling pathway participates in various pathological changes in RA, including synovial inflammation, cartilage damage, bone erosion, and synovial pannus formation [41]. Network pharmacology analysis of the crucial targets for RA treatment was performed for the first time and the results showed that PI3K/Akt and MAPK signaling pathways were among the top ten. Akt and Erk1/2 played key roles in cell proliferation and migration and our study showed that prisitmerin could down-regulate p-Akt and p-Erk, therefore the impact of pristimerin on these crucial proteins was evaluated (Figure 4). Moreover, TNF-α is an overexpressed pro-inflammatory cytokine that would aggravate the pathological states in RA. Depending on our previous research, the terpenoids isolated from the ethyl acetate extract of *Celastrus orbiculatus* Thunb. exhibited good binding affinity with TNF-α, which may be responsible for the therapeutic effects of COE for RA treatment [42]. Molecular docking between pristimerin and TNF-α was also analyzed (Figure 4E and Table S2). According to our work, pristimerin could inhibit the proliferation and migration of MH7A cells and the expression of p-Akt and p-Erk1/2 proteins (Figure 1 or Figure 4A–D). Furthermore, our findings demonstrated that pristimerin exerted anti-RA effects on AIA rats (Figure 5 and Figure 6).

Alterations in protein expression and signaling pathways would surely exert impact on the downstream metabolites, therefore an untargeted metabolomics approach based on UPLC-LQT-orbitrap-MS was employed to capture the changes in endogenous metabolites and metabolic pathways of pristimerin-treated MH7A cells. According to pathway enrichment and topology analysis with KEGG, we found that pristimerin treatment could significantly affect multiple metabolic pathways especially lipid, glutathione and amino acid metabolism (Figure 2 and Figure 3, Table S1). 

Firstly, a series of LysoPCs and LysoPEs were found to be reduced in MH7A cells after pristimerin treatment (Figure 2B and Table S1). LysoPCs, hydrolysis products of membrane PC by phospholipase A2 (PLA2), played key roles in the pathogenesis of inflammatory diseases and the PC/LysoPC ratio in the synovial fluid and plasma of RA patients correlated well with the disease activity, which may act as an indicator of early RA [43]. Focus on lipid metabolism in RA treatment has been closely associated with the finding that RA patients have an increased risk of cardiovascular events during disease progression and studies have reported the elevated cholesterol and lipoprotein levels in synovial fluid of RA patients [44]. RA synovium reside in an oxygen-deprived and cytockine-rich microenvironment similar to solid tumor [2]. The chronically activated RA-FLS are instinctively proliferative, invasive and migratory, and a few studies have shown lipid abnormalities in RA-FLS [45]. Inhibitors of phospholipase D, which specifically cleave phosphatidylcholine (PC) into phosphatidic acid (PA) and choline, could significantly reduce the production of IL-6 and IL-8 in RA-FLS, demonstrating the importance of phospholipid metabolism in synovial inflammation [46]. A large-scale clinical lipidomics analysis of serum samples collected from RA patients and healthy controls discovered 36 lipid metabolites associated with disease progression and among them, phosphatidylethanolamine (PE-16:0/18:2), triglyceride (TG-18:0/18:1/18:2) and PE (18:2/18:2) were recognized as specific biomarkers for RA diagnosis [47].

When considering glutathione metabolism, the oxidized glutathione level was downregulated in pristimerin-treated group and the homeostasis of intracellular reduced/oxidized glutathione (GSH/GSSG) is a significant indicator of cell oxidation state (Figure 2B and Table S1). Glutathione, synthesized from glutamate, cysteine, and glycine, is the most abundant intracelluar antioxidants. Oxidative stress plays an important role in RA pathogenesis and excessive generation of reactive oxygen species (ROS) may impair proteins, lipids and nucleotides [48]. MAPKs, PI3K/Akt and NF-κB signaling pathways can be activated by ROS in RA [49]. One of the major routes for ROS elimination is glutathione oxidation–reduction coupled to NADPH reduction–oxidation [50]. Accordingly, the restoration of glutathione metabolism may be involved in the therapeutic mechanism of pristimerin.

Proteinogenic amino acids including glutamate, glutamine, methionine, threonine, leucine, and phenylalanine were downregulated in pristimerin-treated MH7A cells (Figure 2B and Table S1). As to the downregulation of glutamine, Arra et al. found that glutamine deprivation could cause metabolic reprogramming and alleviation of inflammatory response of chondrocytes, which played an important role in OA [51]. Accordingly, the relationship of glutamine metabolism and inflammatory signaling in MH7A cells deserves further study. Metabolite profiling of RA-FLS and osteoarthritis (OA)-FLS revealed a severe disruption of amino acid metabolism (protein biosynthesis, tyrosine, and catecholamine biosynthesis) in RA-FLS compared with OA-FLS [52]. Although synovial tissues and fluids are thought to be more relevant to physiopathological changes in joints, they are difficult to collect from healthy volunteers [13]. Urine and blood samples are more readily available from healthy volunteers. In a LC-MS-based metabolomics study of RA patients, primary Sjogren’s syndrome (pSS) patients and healthy volunteers, RA patients were found to have relatively higher levels of 4-methoxyphenylacetic acid, leucine, phenylalanine, and glutamate, and the former three metabolites were designated as specific biomarkers for RA [53]. However, the validation of these biomarkers requires much larger sample size and targeted metabolomics approach.

## 5. Conclusions

In the present study, a combined UPLC-LTQ-Orbitrap-based metabolomics and network pharmacology analysis method was first employed to reveal the potential mechanism of pristimerin on MH7A cells. Phenotypic assays, network pharmacology analysis and biochemical tests showed that pristimerin may inhibit MH7A proliferation and migration by regulating Akt and Erk1/2 proteins. Metabolomics analysis demonstrated that pristimerin induced alterations of 20 differential metabolites, which were closely related to phospholipid biosynthesis, fatty acid biosynthesis, glutathione metabolism, and amino acid metabolism. In vivo and in silico studies demonstrated that the anti-RA activity of pristimerin were mediated through the MAPK/Erk1/2, PI3K/Akt pathways and directing binding with TNF-α. Collectively, our study indicated that pristimerin may offer a safe and effective treatment against the development and progression of RA, and the strategy used in our study could be applied to discovery of novel anti-RA drugs.

## Data Availability

The data presented in this study are available in article and Supplementary Materials.

## Supplementary Materials

The following supporting information can be downloaded at: https://www.mdpi.com/article/10.3390/metabo12090839/s1. Table S1: The identification results of potential biomarkers; Table S2: Detailed intermolecular binding interactions of pristimerin with tumor necrosis factor-α (TNF-α); Figure S1: The representative total ion chromatograms (TICs) of the control group in the positive (A) and negative (B) mode and pristimerin-treated group in the positive (C) and negative (D) mode. Figure S2: PCA scores plots based on global metabolic profiles of TNF-α-stimulated MH7A cells with or without pristimerin treatment in the positive (A) and negative (B) modes with following statistics parameters: R^2^X = 0.654, Q^2^ = 0.353 (A); R^2^X = 0.505, Q^2^ = −0.101 (B).

## References

[1] Almutairi K., Nossent J., Preen D., Keen H., Inderjeeth C. (2021). The global prevalence of rheumatoid arthritis: A meta-analysis based on a systematic review. Rheumatol. Int..

[2] Bustamante M.F., Garcia-Carbonell R., Whisenant K.D., Guma M. (2017). Fibroblast-like synoviocyte metabolism in the pathogenesis of rheumatoid arthritis. Arthritis Res. Ther..

[3] Nizer W., Ferraz A.C., Moraes T.F.S., Lima W.G., Santos J.P.D., Duarte L.P., Ferreira J.M.S., de Brito Magalhães C.L., Vieira-Filho S.A., Andrade A. (2021). Pristimerin isolated from *Salacia crassifolia* (Mart. Ex. Schult.) G. Don. (*Celastraceae*) roots as a potential antibacterial agent against *Staphylococcus aureus*. J. Ethnopharmacol..

[4] Jiang Z., Zhao Y., Zhao Y., Liu Y., Tao L. (2021). Pristimerin synergizes with gemcitabine through abrogating Chk1/53BP1-mediated DNA repair in pancreatic cancer cells. Food Chem. Toxicol. Int. J. Publ. Br. Ind. Biol. Res. Assoc..

[5] Li J.J., Yan Y.Y., Sun H.M., Liu Y., Su C.Y., Chen H.B., Zhang J.Y. (2019). Anti-Cancer Effects of Pristimerin and the Mechanisms: A Critical Review. Front. Pharmacol..

[6] Deng Q., Bai S., Gao W., Tong L. (2015). Pristimerin inhibits angiogenesis in adjuvant-induced arthritic rats by suppressing VEGFR2 signaling pathways. Int. Immunopharmacol..

[7] Yousef B.A., Hassan H.M., Zhang L.Y., Jiang Z.Z. (2017). Anticancer Potential and Molecular Targets of Pristimerin: A Mini—Review. Curr. Cancer Drug Targets.

[8] Zhao Q., Liu Y., Zhong J., Bi Y., Liu Y., Ren Z., Li X., Jia J., Yu M., Yu X. (2019). Pristimerin induces apoptosis and autophagy via activation of ROS/ASK1/JNK pathway in human breast cancer in vitro and in vivo. Cell Death Discov..

[9] Tong L., Nanjundaiah S.M., Venkatesha S.H., Astry B., Yu H., Moudgil K.D. (2014). Pristimerin, a naturally occurring triterpenoid, protects against autoimmune arthritis by modulating the cellular and soluble immune mediators of inflammation and tissue damage. Clin. Immunol..

[10] Bai S., Deng W.G.Q., Lin X., Zheng J., Chen Y., Tong L. (2020). Pristimerin Inhibits Adjuvant Arthritis Fibroblast Like Synoviocytes Cell Proliferation through Cell Cycle Arrest and Induction of Apoptosisa. Indian J. Pharm. Sci..

[11] Achudhan D., Liu S.C., Lin Y.Y., Huang C.C., Tsai C.H., Ko C.Y., Chiang I.P., Kuo Y.H., Tang C.H. (2021). Antcin K Inhibits TNF-α, IL-1β and IL-8 Expression in Synovial Fibroblasts and Ameliorates Cartilage Degradation: Implications for the Treatment of Rheumatoid Arthritis. Front. Immunol..

[12] Jing M., Yang J., Zhang L., Liu J., Xu S., Wang M., Zhang L., Sun Y., Yan W., Hou G. (2021). Celastrol inhibits rheumatoid arthritis through the ROS-NF-κB-NLRP3 inflammasome axis. Int. Immunopharmacol..

[13] Menni C., Zierer J., Valdes A.M., Spector T.D. (2017). Mixing omics: Combining genetics and metabolomics to study rheumatic diseases. Nat. Rev. Rheumatol..

[14] Da Silva G.H.R., Mendes L.F., de Carvalho F.V., de Paula E., Duarte I.F. (2022). Comparative Metabolomics Study of the Impact of Articaine and Lidocaine on the Metabolism of SH-SY5Y Neuronal Cells. Metabolites.

[15] Liu J., Luo X., Guo R., Jing W., Lu H. (2020). Cell Metabolomics Reveals Berberine-Inhibited Pancreatic Cancer Cell Viability and Metastasis by Regulating Citrate Metabolism. J. Proteome Res..

[16] Müller-Ladner U., Ospelt C., Gay S., Distler O., Pap T. (2007). Cells of the synovium in rheumatoid arthritis. Synovial fibroblasts. Arthritis Res. Ther..

[17] Nygaard G., Firestein G.S. (2020). Restoring synovial homeostasis in rheumatoid arthritis by targeting fibroblast-like synoviocytes. Nat. Rev. Rheumatol..

[18] Li T., Wei Z., Kuang H. (2021). UPLC-orbitrap-MS-based metabolic profiling of HaCaT cells exposed to withanolides extracted from Datura metel.L: Insights from an untargeted metabolomics. J. Pharm. Biomed. Anal..

[19] Lv M., Chen J., Gao Y., Sun J., Zhang Q., Zhang M., Xu F., Zhang Z. (2015). Metabolomics based on liquid chromatography with mass spectrometry reveals the chemical difference in the stems and roots derived from Ephedra sinica. J. Sep. Sci..

[20] Pang Z., Chong J., Zhou G., de Lima Morais D.A., Chang L., Barrette M., Gauthier C., Jacques P., Li S., Xia J. (2021). MetaboAnalyst 5.0: Narrowing the gap between raw spectra and functional insights. Nucleic Acids Res..

[21] Bailly C., Vergoten G. (2022). Japonicone A and related dimeric sesquiterpene lactones: Molecular targets and mechanisms of anticancer activity. Off. J. Eur. Histamine Res. Soc..

[22] Rigsby R.E., Parker A.B. (2016). Using the PyMOL application to reinforce visual understanding of protein structure. Biochem. Mol. Biol. Educ..

[23] Cui P., Qu F., Sreeharsha N., Sharma S., Mishra A., Gubbiyappa S.K. (2020). Antiarthritic effect of chitosan nanoparticle loaded with embelin against adjuvant-induced arthritis in Wistar rats. IUBMB Life.

[24] Moases Ghaffary E., Abtahi Froushani S.M. (2020). Immunomodulatory benefits of mesenchymal stem cells treated with Caffeine in adjuvant-induced arthritis. Life Sci..

[25] Luo S., Li H., Liu J., Xie X., Wan Z., Wang Y., Zhao Z., Wu X., Li X., Yang M. (2020). Andrographolide ameliorates oxidative stress, inflammation and histological outcome in complete Freund’s adjuvant-induced arthritis. Chem. Biol. Interact..

[26] Scherer H.U., Häupl T., Burmester G.R. (2020). The etiology of rheumatoid arthritis. J. Autoimmun..

[27] Kedia A.K., Mohansundaram K., Goyal M., Ravindran V. (2021). Safety of long-term use of four common conventional disease modifying anti-rheumatic drugs in rheumatoid arthritis. J. R. Coll. Physicians Edinb..

[28] Singh J.A. (2022). Treatment Guidelines in Rheumatoid Arthritis. Rheum. Dis. Clin. North Am..

[29] Smolen J.S., Aletaha D. (2015). Rheumatoid arthritis therapy reappraisal: Strategies, opportunities and challenges. Nat. Rev. Rheumatol..

[30] Yi O., Lin Y., Hu M., Hu S., Su Z., Liao J., Liu B., Liu L., Cai X. (2022). Lactate metabolism in rheumatoid arthritis: Pathogenic mechanisms and therapeutic intervention with natural compounds. Phytomed. Int. J. Phytother. Phytopharm..

[31] Chen R.Z., Yang F., Zhang M., Sun Z.G., Zhang N. (2021). Cellular and Molecular Mechanisms of Pristimerin in Cancer Therapy: Recent Advances. Front. Oncol..

[32] Renda G., Gökkaya İ., Şöhretoğlu D. (2022). Immunomodulatory properties of triterpenes. Phytochem. Rev. Proc. Phytochem. Soc. Eur..

[33] Xu S., Lyu L., Zhu H., Huang X., Xu W., Xu W., Feng Y., Fan Y. (2021). Serum Metabolome Mediates the Antiobesity Effect of Celastrol-Induced Gut Microbial Alterations. J. Proteome Res..

[34] Zhang X.W., Chen Y.T., Feng X., Li L.Y., Song K.W., Sun Y.P., Zhang G.H., Zhang L.T. (2022). A comprehensive study of celastrol metabolism in vivo and in vitro using ultra-high-performance liquid chromatography coupled with hybrid triple quadrupole time-of-flight mass spectrometry. J. Sep. Sci..

[35] Abu Bakar M.H., Nor Shahril N.S., Mohamad Khalid M.S.F., Mohammad S., Shariff K.A., Karunakaran T., Mohd Salleh R., Mohamad Rosdi M.N. (2022). Celastrol alleviates high-fat diet-induced obesity via enhanced muscle glucose utilization and mitochondrial oxidative metabolism-mediated upregulation of pyruvate dehydrogenase complex. Toxicol. Appl. Pharmacol..

[36] Zhang T., Zhao Q., Xiao X., Yang R., Hu D., Zhu X., Gonzalez F.J., Li F. (2019). Modulation of Lipid Metabolism by Celastrol. J. Proteome Res..

[37] Hong W., Park J., Yun W., Kang P.J., Son D., Jang J., Kim I.Y., You S. (2018). Inhibitory effect of celastrol on adipogenic differentiation of human adipose-derived stem cells. Biochem. Biophys. Res. Commun..

[38] Yang L., Liu R., Fang Y., He J. (2021). Anti-inflammatory effect of phenylpropanoids from Dendropanax dentiger in TNF-α-induced MH7A cells via inhibition of NF-κB, Akt and JNK signaling pathways. Int. Immunopharmacol..

[39] Li T.P., Zhang A.H., Miao J.H., Sun H., Yan G.L., Wu F.F., Wang X.J. (2019). Applications and potential mechanisms of herbal medicines for rheumatoid arthritis treatment: A systematic review. RSC Adv..

[40] Liu Z., Guo S., Dong Q. (2020). Nobiletin suppresses IL-21/IL-21 receptor-mediated inflammatory response in MH7A fibroblast-like synoviocytes (FLS): An implication in rheumatoid arthritis. Eur. J. Pharmacol..

[41] Sun K., Luo J., Guo J., Yao X., Jing X., Guo F. (2020). The PI3K/AKT/mTOR signaling pathway in osteoarthritis: A narrative review. Osteoarthr. Cartil..

[42] Lv M., Liang Q., Wan X., Wang Z., Qian Y., Xiang J., Luo Z., Ni T., Jiang W., Wang W. (2022). Metabolomics and molecular docking-directed antiarthritic study of the ethyl acetate extract from Celastrus orbiculatus Thunb. J. Ethnopharmacol..

[43] Fuchs B., Schiller J., Wagner U., Häntzschel H., Arnold K. (2005). The phosphatidylcholine/lysophosphatidylcholine ratio in human plasma is an indicator of the severity of rheumatoid arthritis: Investigations by 31P NMR and MALDI-TOF MS. Clin. Biochem..

[44] McGrath C.M., Young S.P. (2015). Lipid and Metabolic Changes in Rheumatoid Arthritis. Curr. Rheumatol. Rep..

[45] Falconer J., Murphy A.N., Young S.P., Clark A.R., Tiziani S., Guma M., Buckley C.D. (2018). Review: Synovial Cell Metabolism and Chronic Inflammation in Rheumatoid Arthritis. Arthritis Rheumatol..

[46] Friday S.C., Fox D.A. (2016). Phospholipase D enzymes facilitate IL-17- and TNFα-induced expression of proinflammatory genes in rheumatoid arthritis synovial fibroblasts (RASF). Immunol. Lett..

[47] Zhou G., Lu J., Xu T., Lu Y., Chen W., Wang J., Ke M., Shen Q., Zhu Y., Shan J. (2021). Clinical lipidomics analysis reveals biomarkers of lipid peroxidation in serum from patients with rheumatoid arthritis. Microchem. J..

[48] Alisik M., Alisik T., Nacir B., Neselioglu S., Genc-Isik I., Koyuncu P., Erel O. (2021). Erythrocyte reduced/oxidized glutathione and serum thiol/disulfide homeostasis in patients with rheumatoid arthritis. Clin. Biochem..

[49] Phull A.R., Nasir B., Haq I.U., Kim S.J. (2018). Oxidative stress, consequences and ROS mediated cellular signaling in rheumatoid arthritis. Chem. Biol. Interact..

[50] Pan T., Han D., Xu Y., Peng W., Bai L., Zhou X., He H. (2021). LC-MS Based Metabolomics Study of the Effects of EGCG on A549 Cells. Front. Pharmacol..

[51] Arra M., Swarnkar G., Adapala N.S., Naqvi S.K., Cai L., Rai M.F., Singamaneni S., Mbalaviele G., Brophy R., Abu-Amer Y. (2022). Glutamine metabolism modulates chondrocyte inflammatory response. Elife.

[52] Ahn J.K., Kim S., Hwang J., Kim J., Kim K.H., Cha H.S. (2016). GC/TOF-MS-based metabolomic profiling in cultured fibroblast-like synoviocytes from rheumatoid arthritis. Jt. Bone Spine.

[53] Li J., Che N., Xu L., Zhang Q., Wang Q., Tan W., Zhang M. (2018). LC-MS-based serum metabolomics reveals a distinctive signature in patients with rheumatoid arthritis. Clin. Rheumatol..

