# Supplementary material for: UPLC-LTQ-Orbitrap-Based Cell Metabolomics and Network Pharmacology Analysis to Reveal the Potential Antiarthritic Effects of Pristimerin: In Vitro, In Silico and In Vivo Study
Source: Metabolites. 2022 Sep 5;12(9):839. doi: 10.3390/metabo12090839 (PMC9505172; doi:10.3390/metabo12090839)
Supplement: Supplementary file 1 [file metabolites-12-00839-s001.zip › metabolites-1892346-supplementary.pdf]

Supporting Information

UPLC-LTQ-Orbitrap-Based Cell Metabolomics and Network Pharmacology Analysis to Reveal the Potential Antiarthritic Effects of Pristimerin: In Vitro, in Silico and In Vivo Study

Mengying Lv [1,2,*,†], Qiaoling Liang [1,2,†], Zhaoyong Luo [1,2,†], Bo Han [3], Tengyang Ni [1,2], Yang Wang [1,2], Li Tao [1,2], Weiting Lyu [4], Jie Xiang [1,*] and Yanqing Liu [1,2,*]

1 Department of Pharmacy, Institute of Translational Medicine, Medical College, Yangzhou University, Yangzhou 225001, China
2 The Key Laboratory of Syndrome Differentiation and Treatment of Gastric Cancer of the State Administration of Traditional Chinese Medicine, Yangzhou 225001, China
3 Key Laboratory of Xinjiang Phytomedicine Resource and Utilization, School of Pharmacy, Ministry of Education, Shihezi University, Shihezi 832002, China
4 Department of Medicinal Chemistry, Rutgers University, Piscataway, NJ 08854, USA
* Correspondence: lvmengying1988114@163.com (M.L.); jxiang@yzu.edu.cn (J.X.); liuyq@yzu.edu.cn (Y.L.)
† These authors contributed equally to this work.

Table S1. The identification results of potential biomarkers.

Name	Formula	Retention time (min)	Measured (m/z)	ESI ionization mode	MS/MS fragments	Trend	Metabolic pathway
N-Methylnicotinamide	$C_7H_8N_2O$	0.636	137.0707	+	119.0602	↓	Amino acid metabolism
Glutamate	$C_5H_9NO_4$	0.660	148.0600	+	130.0494	↓	Amino acid metabolism
Methionine	$C_5H_{11}NO_2S$	0.843	150.0579	+	104.0526	↓	Amino acid metabolism
Oxidized glutathione	$C_{20}H_{32}N_6O_{12}S_2$	0.845	613.1581	+	484.1155,130.0495	↓	Glutathione metabolism
Leucine*	$C_6H_{13}NO_2$	0.659	132.0763	+	115.0535	↓	Amino acid metabolism
Phenylalanine	$C_9H_{11}NO_2$	1.026	166.0859	+	120.0803,103.0538,95.0487,77.0381	↓	Phenylalanine metabolism
LysoPE(20:4)	$C_{25}H_{44}NO_7P$	6.859	502.2919	+	484.2820,441.2394,361.2733	↓	Glycerophospholipid catabolism
LysoPC(16:0)	$C_{24}H_{50}NO_7P$	7.141	496.3386	+	478.3275,184.0728	↓	Glycerophospholipid catabolism
LysoPE(16:0)	$C_{21}H_{44}NO_7P$	7.333	454.2918	+	313.2727	↓	Glycerophospholipid catabolism
LysoPE(18:1)	$C_{23}H_{46}NO_7P$	7.600	480.3074	+	462.2969,339.2886	↓	Glycerophospholipid catabolism
LysoPC(18:1)	$C_{26}H_{52}NO_7P$	7.636	522.3541	+	504.3451,184.0732,104.1068	↓	Glycerophospholipid catabolism
LysoPE(18:0)	$C_{23}H_{48}NO_7P$	8.517	482.3225	+	464.3125,341.3037	↓	Glycerophospholipid catabolism
LysoPC(18:0)	$C_{26}H_{54}NO_7P$	8.552	524.3701	+	506.3608,341.3048,184.0732,104.1067	↑	Glycerophospholipid catabolism
Myristic acid	$C_{14}H_{28}O_2$	10.195	227.2019	-	209.1909	↑	Fatty acid metabolism
Ceramide(d18:0/12:0)	$C_{30}H_{61}NO_3$	11.031	484.4708	+	466.4629,256.2628	↑	Lipid metabolism
Palmitic acid	$C_{16}H_{32}O_2$	11.701	255.2327	-	237.2212	↑	Fatty acid metabolism
Ceramide(d18:0/14:0)	$C_{32}H_{65}NO_3$	12.553	512.5020	+	494.4912,256.2632	↑	Lipid metabolism
Stearic acid	$C_{18}H_{36}O_2$	13.847	283.2639	-	265.2521	↑	Fatty acid metabolism
Ceramide(d18:0/16:0)	$C_{34}H_{69}NO_3$	15.060	540.5330	+	522.5965,284.2942,256.2632	↓	Lipid metabolism
SM(18:1/16:0)	$C_{39}H_{79}N_2O_6P$	16.436	703.5729	+	502.4972,184.0728	↓	Lipid metabolism

Table S2. Detailed intermolecular binding interactions of pristimerin with tumor necrosis factor-α (TNF-α)

Protein	Binding energy (Kcal/mol)	Interacting residues
tumor necrosis factor-α (TNF-α)	-9.5	Tyr59, Tyr119, Ile 155, Leu 120, Gly121, Tyr59, Gly121, His15, Tyr119, Ser60 and Tyr151

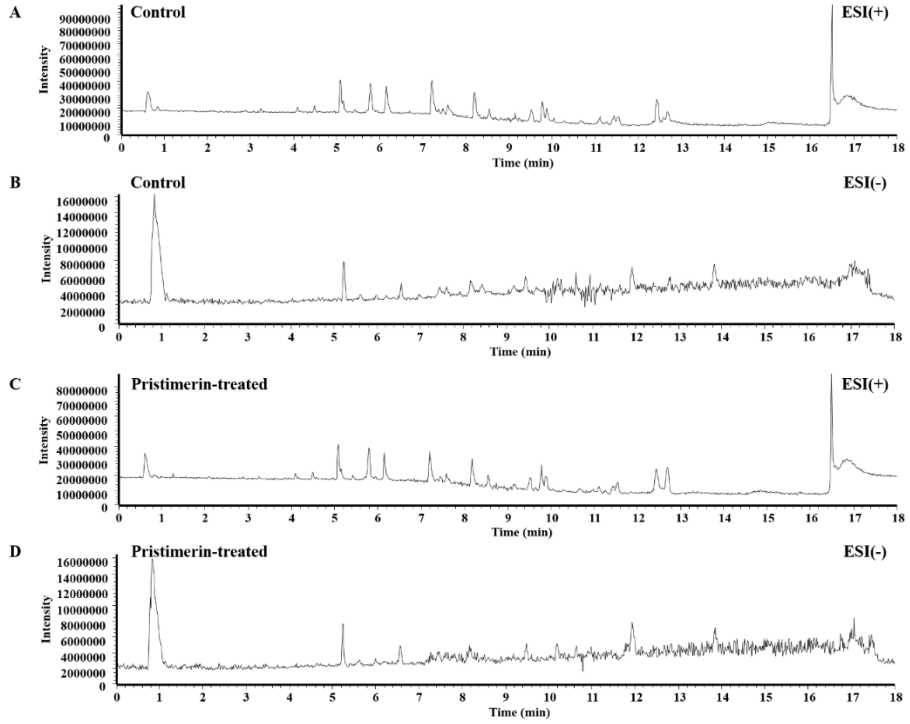

Figure S1. The representative total ion chromatograms (TICs) of the control group in the positive (**A**) and negative (**B**) mode and pristimerin-treated group in the positive (**C**) and negative (**D**) mode.

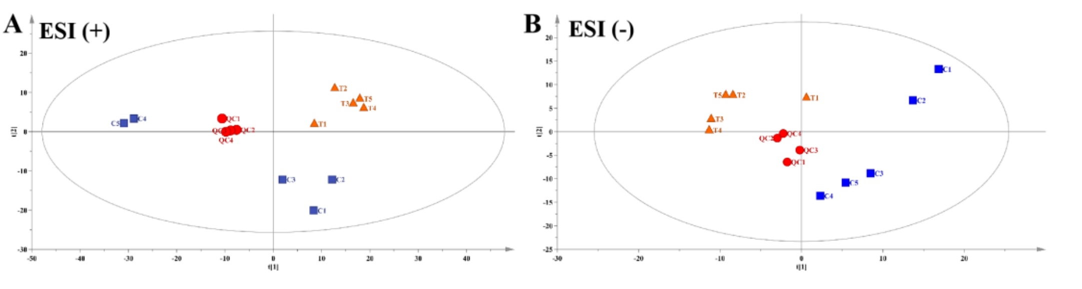

Figure S2. PCA scores plots based on global metabolic profiles of TNF-α-stimulated MH7A cells with or without pristimerin treatment in the positive (A) and negative (B) modes with following statistics

parameters: $R^2X=0.654$, $Q^2=0.353$ (A); $R^2X=0.505$, $Q^2=-0.101$ (B). The letter "C" refers to TNF-α-stimulated MH7A cells without pristimerin treatment and the letter "T" refers to TNF-α-stimulated MH7A cells with pristimerin treatment.
